# Case of Rattlesnake Bite

**Published:** 1861-10

**Authors:** L. S. Ham

**Affiliations:** South Bend, Ind.


ART. V.—*Case of Rattlesnake Bite; by* L. S. HAM, *of South Bend, Ind.*

DR. S. W. MITCHELL has an article on the Bite of the Rattlesnake, in the March number of the *North American Medico-Chiurgical Review*. It appears that Dr. M. had experimented very largely on the treatment of bites by the rattlesnake. He has kept snakes, dogs, cats, rabbits, pigeons, &c., for the purpose of experimenting on this important subject.

The Doctor seems to have experimented very carefully, and judged very candidly on the result of his observations, with the different remedies, which he tested upon different animals. He dwelt rather lengthily on those remedies which seem to share the largest portion of confidence in the mind of the Profession at the present time. He dwells very particularly on Prof. BRAINARD's treatment, on BIBRON's ANTIDOTE. His opinions of BIBRON's ANTIDOTE may be gleaned from the following extracts:

"The experiments here related, and the general results of the use of Prof. BIBRON's ANTIDOTE are, on the whole, so discouraging as to render probable that it is in reality no more valuable than other agents which have once enjoyed an equal reputation. While expressing this opinion, founded chiefly on my own experience, I cannot help feeling that it is impossible to settle the question definitely without further and longer experience in the form of human cases of venom poisoning, which should be studied with care by the light which I have endeavored to throw upon the subject—reference being had to the number of fang marks, the size of the snake," &c.

I write for the single purpose of giving the Profession the result of a single case which came under my care about one year since. The case which I will now give, has reference to the *two points* which Dr. M. thinks should have "further and longer experience in the form of human cases," to wit: *the number of fang marks and the size of the snake.* I copy the following from my case book:

"September 16, 1860, Mr. David Lobdell came to my office at 1½ o'clock P. M., in great alarm and agitation from having been bitten by a rattlesnake. At 10 o'clock A. M., to-day, Mr. Lobdell with two young friends were hunting prairie chicken on the Kankakee marsh, five miles from town. Mr. Lobdell stooped to pluck a most beautiful flower, and at that moment received a full blow from a concealed rattlesnake which measured 3 feet 2½ inches. The blow was received on the right hand, in that triangular fleshy portion which lies between the thumb and the index finger. By drawing a line from the second joint of the thumb and the third joint of the index finger, and one-fourth of an inch above or back of that line, will give you the exact locality of the wound. Both fangs entered full length, and the snake had to be shaken from his hold. One of the friends killed the snake, and the other, as he thought and intended, excised the part; but he only cut skin deep. After having killed the snake they bound a portion of the flesh upon the wound and ligated the arm above the elbow; in which condition I saw the patient three and one-half hours after the infliction of the wound. Hand much swollen—swelling extends nearly to the elbow. Hand and lower part of the fore-arm dark and mottled. Has walked five miles to town since the bite; is very warm; pulse 116; has vomited several times by the way, and has quite an indistinctness of vision. Washed the hand, and passed a very fine silver probe to the bottom of the fang wounds, and find each of them a full half inch deeper than the incision of the parts. Gave four ounces of good brandy at a single dose; next injected tinc. iodine into the fang wounds, and gave valerinate morphine one-half grain, and wrapped the whole hand in a cloth saturated with tinc. iodine; continued brandy in ounce doses every half hour.

At three P. M. the swelling of the hand and of the fore-arm had increased very rapidly; now extends nearly to the shoulder. At precisely 3½ o'clock P. M. commenced to give Bibron's Antidote in 10 drop doses every two hours. It is composed of

℞ Potassi Iodidi, - - - - - - - - - - - gr. iv
Hydr. Bichlor, - - - - - - - - - - - gr. ii
Bromine, - - - - - - - - - - - - - ʒ v
M

Medium dose, 10 gtt. in a little brandy.

Six P. M. retains the antidote well on the stomach. 10½ P. M. swelling arrested for the time, since antidote was first administered; symptoms same; treatment same.

Monday, Sept. 17th, 5 A. M. Symptoms same; treatment continued 10 A. M., rejects antidote in any and every quantity; give

℞ Val. Morph. - - - - - - - - - - - gr. iv
Pulv. Camph. - - - - - - - - - - - - gr. i
Cal. - - - - - - - - - - - - - - - gr. i
M

One to be taken every hour.

Twelve M. Vomiting continues; swelling of the hand and of the whole arm increases with fearful rapidity—extends to the chest and neck. 4 P. M. Not vomited since 1½ o'clock; commence to give antidote again in five drop doses every hour; swelling ceased to progress; hand and fore-arm enormously swollen; omit morphine; signs of gangrene about the hand; apply yeast poultice. 11 o'clock P. M. Same as at 4; continue treatment; bears antidote well.

Tuesday, 18th, 5 A. M. Vomit as soon as antidote is swallowed; rejects it at once; suspend antidote; give morphine again and milk punch.—11½ A. M. The whole trunk and neck enormously swollen; has had a free dejection by the bowels; treatment continued. 5 P. M. Swelling on the increase about the neck and head; comatose; pulse 140; feeble; great nervous prostration; continued low muttering; omit morphine; continue punch; add

℞ Strychnine, - - - - - - - - - - - - - gr. i

Nitric Acid, - - - - . - - - - - - - - ʒ i

Tinc. Opii. - - - - - - - - - - - - ʒ ii

Syr. Simp. - - - - - - - - - - - - ℥ iii

M

Teaspoonful every four hours.

10½ P. M. Has not vomited since he took the above; commence antidote again in 5 drop doses, every hour; swelling has been on the increase since he omitted the antidote.

Wednesday, 19th, 4 A. M. Swelling ceased to increase since twelve last night; treatment continued. 12 M. Pulse 118; capillary circulation better; retains medicine well; takes nourishment and punch freely; swelling of head and neck begin to diminish. 9 P. M. Patient same; continue treatment.

Thursday, 20th, 6 A. M. Patient convalescent; continued the use of the antidote in 4 drop doses till the 23d; continued the punch and the following tonic mixture:

| | | |
|---|---|---|
| ℞ | Tinc. Cinchona, } Tinc. Columbo, } - - - - - - - a a | ℥ i |
| | Tinc. Nuxvomica, - - - - - - - - - - | ʒ i |
| | | M |

In teaspoonful doses, every eight hours, until the 9th day of October, when the patient was discharged cured.

By the way, I should here add that Dr. HIGINBOTHAM was united with myself in the treament of the case, and that Drs. HUMPHREYS and MYERS were in consultation at different times during the first three days of the disease, and that we received valuable advice from each of these gentlemen.

The foregoing case shows several important facts in the reception, the progress and the treatment of this important class of maladies.

1.—The fang wounds were deep, and we have every reason to conclude that all was done that the animal was capable of doing by way of imparting poison to the system.

2.—The snake was one of the very largest of the Massasaugus variety.

3.—The poison had had its local and constitutional effects before treatment was commenced, as shown by the swelling of the hand, the vomiting, and the indistinctness of vision.

4.—The good effects of the antidote are shown by the suspension of the swelling as soon as it was administered—the increase of the swelling as soon as it was rejected—and the suspension of the swelling again as soon as it could be retained on the stomach, in doses equal to from three to five drops per hour. This was very clearly evinced on two distinct occasions in our case.

5.—Its apparent good effects in my hands, and under all the circumstances are such as to give me great confidence in its therapeutic powers in this class of cases. Yet, I am aware that it would take many cases, and further tests, to fully establish its claim to full confidence in the mind of the Profession.

